# Feedback facilitation by adenosine A_2A_ receptors of ATP release from mouse hippocampal nerve terminals

**DOI:** 10.1007/s11302-023-09937-y

**Published:** 2023-03-31

**Authors:** Francisco Q. Gonçalves, Pedro Valada, Marco Matos, Rodrigo A. Cunha, Angelo R. Tomé

**Affiliations:** 1 CNC-Center for Neuroscience and Cell Biology, University of Coimbra, 3004-504 Coimbra, Portugal; 2 FMUC - Faculty of Medicine, University of Coimbra, 3004-504 Coimbra, Portugal; 3 Department of Life Sciences, Faculty of Sciences and Technology, University of Coimbra, 3004-517 Coimbra, Portugal

**Keywords:** ATP, release, adenosine, A_1_ receptor, A_2A_ receptor, ecto-5’-nucleotidase, CD73, hippocampus, nerve terminals, synaptosomes

## Abstract

The adenosine modulation system is mostly composed by inhibitory A_1_ receptors (A_1_R) and the less abundant facilitatory A_2A_ receptors (A_2A_R), the latter selectively engaged at high frequency stimulation associated with synaptic plasticity processes in the hippocampus. A_2A_R are activated by adenosine originated from extracellular ATP through ecto-5’-nucleotidase or CD73-mediated catabolism. Using hippocampal synaptosomes, we now investigated how adenosine receptors modulate the synaptic release of ATP. The A_2A_R agonist CGS21680 (10-100 nM) enhanced the K^+^-evoked release of ATP, whereas both SCH58261 and the CD73 inhibitor α,β-methylene ADP (100 μM) decreased ATP release; all these effects were abolished in forebrain A_2A_R knockout mice. The A_1_R agonist CPA (10-100 nM) inhibited ATP release, whereas the A_1_R antagonist DPCPX (100 nM) was devoid of effects. The presence of SCH58261 potentiated CPA-mediated ATP release and uncovered a facilitatory effect of DPCPX. Overall, these findings indicate that ATP release is predominantly controlled by A_2A_R, which are involved in an apparent feedback loop of A_2A_R-mediated increased ATP release together with dampening of A_1_R-mediated inhibition. This study is a tribute to María Teresa Miras-Portugal.

## Introduction

ATP is a multifactorial signaling molecule in the brain, involved in the communication between glia cells as well as in the bidirectional communication between glia and neurons (reviewed in [1]). Extracellular ATP is also produced upon synaptic activity in accordance with the accumulation of ATP in synaptic vesicles and its release with different neurotransmitters (e.g. [2, 3]), namely in the hippocampus [4–6], a brain region where synaptic plasticity processes are proposed to encode reference memory traits [7]. Although hippocampal synapses are endowed with different ATP-activated P_2_ receptors (e.g. [8]), the most evident role of extracellular ATP is to be a substrate for the action of ecto-nucleotidases, regulated by ecto-5’-nucleotidase or CD73 [9], to form extracellular adenosine to selectively activate adenosine A_2A_ receptors (A_2A_R) [10–12]. A_2A_R are selectively engaged to control synaptic plasticity processes [13–17] and control memory and neurodegeneration (reviewed in [18]).

The adenosine neuromodulation system is a classical neuromodulation system, with a powerful inhibitory effect operated by A_1_R and a selective recruitment of A_2A_R to control synaptic plasticity [18], which involve a discrete facilitation of neurotransmitter release [19–21], the attenuation of the predominant A_1_R-mediated inhibition [19, 22, 23] and a post-synaptic facilitation of NMDA receptor-mediated responses [14, 24]. Since ATP release selectively occurs at high frequency simulation [4, 25] and selectively feeds A_2A_R to control synaptic plasticity processes, we now explored if A_2A_R control of ATP release from nerve terminals is a putative feedback loop involving CD73-mediated formation of adenosine from released ATP to activate A_2A_R-mediated facilitation of ATP release, as occurs for astrocytic ATP release [26]. Furthermore, we also aimed at understanding if ATP release is affected by the A_1_R-mediated inhibitory system, which robustly inhibits the release of classical neurotransmitters.

## Methods

### Animals

We used 32 male and female mice (20.8±0.2 g, 8-10 weeks old) from our inbred colony of forebrain A_2A_ receptor knockout mice with a C57BL/6 genetic background [11] and wild type C57BL/6 obtained from Charles River (Barcelona, Spain). Mice were housed in collective cages with an enriched environment in HEPA-filtered ventilated racks (n=3-5 per cage) under a controlled environment (12 h light-dark cycle, lights on at 7 AM, and room temperature 22±1°C) with *ad libitum* access to food and water. The study was approved by the Ethical Committee of the Center for Neuroscience and Cell Biology (ORBEA n° 138-2016/15072016), following European Union guidelines (2010/63).

### Preparation of synaptosomes

In spite of their artificial nature, difficulties in experimentally triggering neurotransmitter release with a ‘physiological’ pattern and their heterogeneity, synaptosomes are still the most adequate preparation to unambiguously ascribe mechanisms as occurring presynaptically [27]. Hippocampal synaptosomes (purified synapses) were prepared as previously described [28]. After deep anesthesia under halothane atmosphere, each mouse was decapitated, the two hippocampi were dissected and homogenized in sucrose (0.32 M) solution containing 1 mM EDTA, 10 mM HEPES, 1 mg/mL bovine serum albumin (Sigma), pH 7.4 at 4 °C, supplemented with a protease inhibitor, phenylmethylsulfonyl fluoride (PMSF 0.1 mM), a cocktail of inhibitors of proteases (CLAP 1%, Sigma) and the antioxidant dithiothreitol (1 μM). The homogenate was centrifuged at 3,000 x *g* for 10 min at 4 °C and the resulting supernatant was further centrifuged at 14,000 x *g* for 12 min at 4 °C. The resulting pellet (P2 fraction) was resuspended in 1 mL of a 45% (v/v) Percoll solution in Krebs-HEPES buffer (140 mM NaCl, 5 mM KCl, 25 mM HEPES, 1 mM EDTA, 10 mM glucose; pH 7.4). After centrifugation at 14,000 x *g* for 2 min at 4 °C, the white top layer was collected (synaptosomal fraction), resuspended in 1 mL Krebs-HEPES buffer and further centrifuged at 14,000 x *g* for 2 min at 4 °C. The pellet was then resuspended in Krebs-HEPES solution. The purity of this synaptic fraction has been previously quantified as >95% [28].

### ATP release

The release of ATP was measured on-line using the luciferin-luciferase assay, as previously described [11]. Briefly, a suspension containing synaptosomes, an ATP assay mix (with luciferin and luciferase; from Sigma) and Krebs-HEPES solution was equilibrated at 25 °C during 10 min to ensure the functional recovery of nerve terminals. The suspension was then transferred to a white 96-well plate and measurements were performed in a luminometer (Victor3). After 60 seconds to measure basal ATP outflow, the evoked release of ATP was triggered with 32 mM of KCl (isomolar substitution of NaCl in the Krebs-HEPES solution), a well-established neurochemical strategy to trigger optimal signal-to-noise calcium-dependent vesicular release from synaptosomes without damage to these artificial synaptic structures [27]. The evoked release of ATP was calculated by integration of the area of the peak upon subtraction of the estimated basal ATP outflow [11].

### Pharmacological manipulations

We used the A_2A_R agonist 2-[4-(2-p-carboxyethyl)phenylamino]-5’-N-ethylcarboxamidoadenosine (CGS21680, Tocris) in a selective concentration range (10-100 nM; [29]), the A_2A_R antagonist 5-amino-7-(2-phenylethyl)-2-(2-furyl)-pyrazolo-[4,3-e]-1,2,4 triazolo[1,5-c]pyrimidine (SCH58261; Tocris) at a supra-maximal but selective concentration (50 nM; [14]), the selective A_1_R agonist N^6^-cyclopentyladenosine (CPA, Tocris) in a selective concentration range (10-100 nM; [30]), the A_1_R antagonist 1,3-dipropyl-8-cyclopentylxanthine (DPCPX, Tocris) at a supra-maximal but selective concentration (100 nM [30]) and the ecto-5’-nucleotidase or CD73 inhibitor α,β-methylene ADP (AOPCP, Sigma-Aldrich) at a supra-maximal but selective concentration (100 μM [11]). AOPCP was directly prepared in Krebs solution, whereas all adenosine receptor ligands were prepared as 5 mM stock solutions in dimethylsulfoxide.

### Statistics

The values are presented as mean±S.E.M. The percentage effect of drugs was calculated in each individual experiment and the S.E.M. is relative to the variance of this percentage effect. To test the significance of the effect of drugs *versus* control, a paired Student’s t test was used. When making comparisons from a different set of experiments with control, a one-way analysis of variance (ANOVA) was used, followed by a Dunnett’s test. P < 0.05 was considered to represent a significant difference.

## Results

### A_2A_ receptors increase the evoked release of ATP

**Fig. 1:**
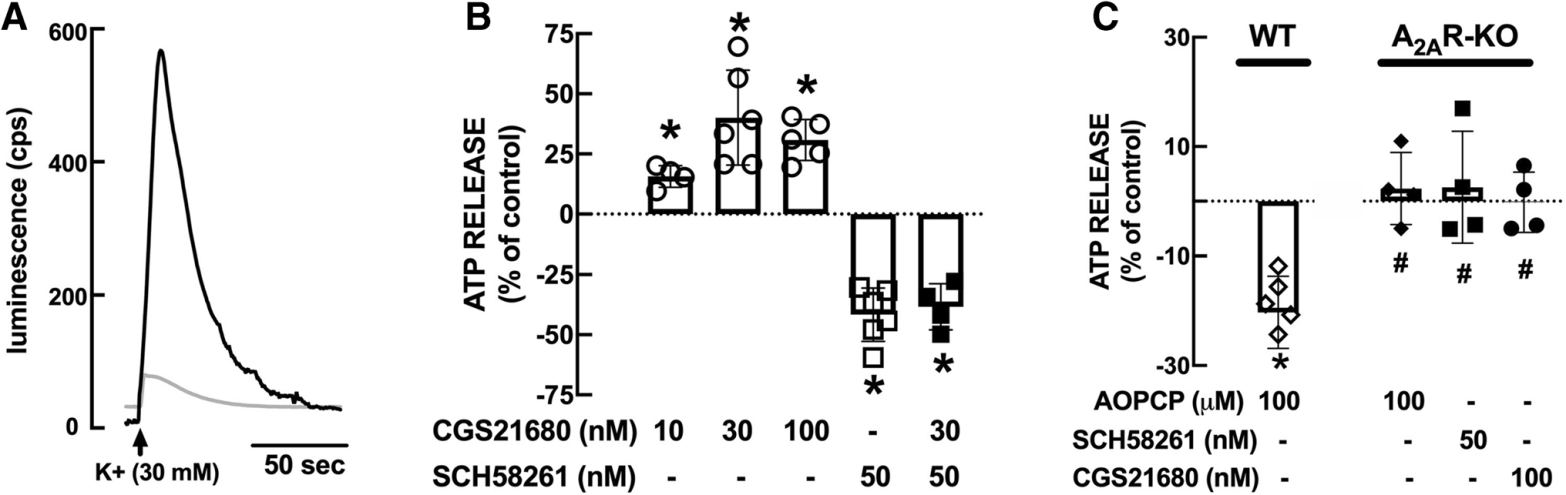
Adenosine A_2A_ receptors (A_2A_R) increase the evoked release of ATP that sustains the activation of A_2A_R through CD73-mediated formation of extracellular adenosine in hippocampal synaptosomes. (**A**) Representative recording of luminescence emitted by luciferase as a measure of extracellular ATP in hippocampal synaptosomes depolarized with addition of KCl (30 mM) in the presence (black) and absence of extracellular calcium (grey), showing that the evoked release of ATP is expected to be vesicular in nature. (**B**) The A_2A_R agonist CGS21680 (10-100 nM) enhanced the evoked release of ATP, whereas the A_2A_R antagonist SCH58261 (50 nM) decreased the evoked release of ATP and prevented any action of CGS21680. (**C**) The ecto-5’-nucleotidase (CD73) inhibitor α,β-methylene ADP (AOPCP, 100 μM) inhibited the evoked release of ATP and neither AOPCP nor A_2A_R ligands modified the evoked ATP release in hippocampal synaptosomes from A_2A_R knockout mice. Data are mean SEM of n=4-6 different mice in (**B,C**). *p<0.05 one-way Student’s t test *vs*. 0%; #p<0.05 two-tailed Student’s t test between genotypes.

The K^+^-induced release of ATP from nerve terminals likely reflects a vesicular release of ATP [2, 5, 31, 32], as now confirmed by the dependency of this evoked ATP release on the presence of extracellular free calcium. Thus, the elevation of extracellular K^+^ to 30 mM to depolarize synaptosomes triggered a release of ATP (Fig. 1A, black line); the average K^+^-evoked ATP release was 21.9±2.3 pmol/mg protein (n=6). This K^+^-induced release of ATP was reduced by over 90% in a Krebs medium without added calcium (n=4; Fig. 1A, grey line).

Since the activation of A_2A_R facilitates the release of different classical neurotransmitters from hippocampal nerve terminals [19, 21, 33], we now tested if A_2A_R activation also facilitated the release of ATP. The A_2A_R agonist CGS21680 increased the evoked release of ATP and this facilitation was larger (p=0.044) at 30 nM CGS21680 (40.1±8.0% facilitation, n=6) than at 10 nM (15.7±2.3%, n=4) and saturated at 100 nM (30.4±4.1%, n=5) (Fig. 1B). The selective A_2A_R antagonist SCH58261 (50 nM) decreased the evoked release of ATP by 41.7±4.5% (n=6), indicating that endogenous adenosine tonically activates A_2A_R to bolster ATP release (Fig. 1B). Furthermore, CGS21680 (30 nM) was devoid of effects (p=0.391; n=4) in the presence of 50 nM SCH58261 (Fig. 1B), further confirming the involvement of A_2A_R in the control of ATP release from hippocampal synaptosomes. Importantly, neither CGS21680 (10-100 nM) nor SCH58261 (50 nM) modified the basal outflow of ATP in the absence of K^+^-induced depolarization (data not shown).

### CD73-mediated ATP-derived adenosine feeds A_2A_ receptors to control ATP release

Since A_2A_R are selectively activated by CD73-mediated extracellular ATP-derived adenosine [10–12, 32, 34], we probed if CD73 was involved in a putative feedback facilitating loop of ATP release from nerve terminals, as previously observed for ATP release from astrocytes [26]. Thus, we tested the impact of the CD73 inhibitor AOPCP on the evoked release of ATP from hippocampal nerve terminals. As shown in Fig. 1C, AOPCP (100 μM) inhibited the K^+^-evoked release of ATP by 20.3±2.7% (n=6) in hippocampal synaptosomes from wild type mice, where AOPCP was devoid of effects (p=0.351; n=4) in hippocampal synaptosomes from A_2A_R knockout mice (Fig. 1C) and did not modify the basal outflow of ATP in the absence of K^+^-induced depolarization in wild type or A_2A_R knockout mice (data not shown). Moreover, the evoked release of ATP from hippocampal synaptosomes from A_2A_R knockout mice was not modified by either 30 nM CGS21680 (p=0.947; n=4) or 50 nM SCH58261 (p=0.653; n=4) (Fig. 1C), further reenforcing a putative feedback modulation role of A_2A_R in bolstering ATP release as a consequence of extracellular ATP-derived adenosine formation.

### A_2A_ receptors dampen A_1_ receptor-mediated inhibition of ATP release

The concluded robust effect of A_2A_R in the control of ATP release is somewhat surprising in view of the discrete impact of A_2A_R in the control of different classical neurotransmitters from hippocampal nerve terminals, such as glutamate [19], GABA [33] or acetylcholine [21]. Since A_2A_R control A_1_R-mediated effects in nerve terminals [19, 22, 23], we next investigated the impact of A_1_R on ATP release and the effect of A_2A_R on this putative A_1_R-mediated modulation of ATP release.

**Fig. 2:**
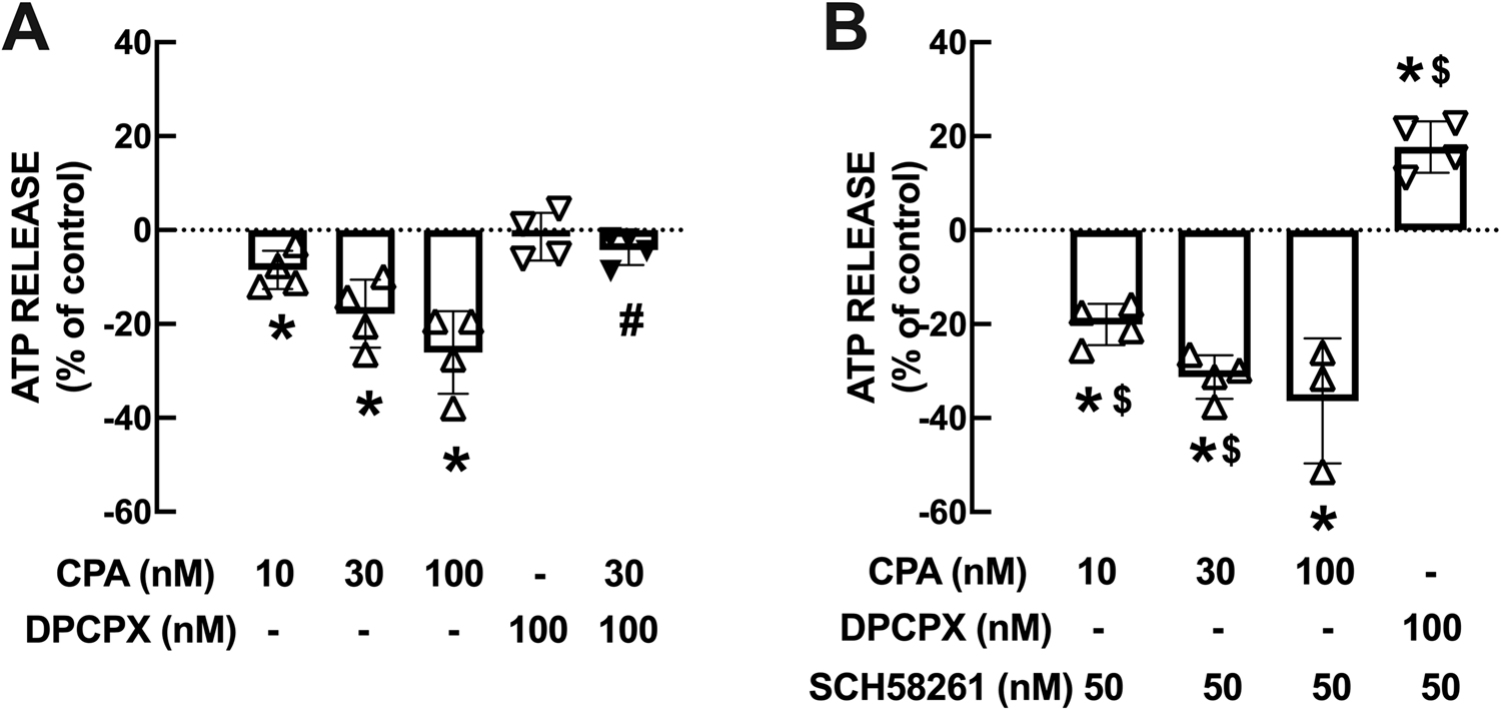
Adenosine A_2A_ receptors dampen the adenosine A_1_ receptor-mediated inhibition of the evoked release of ATP from hippocampal synaptosomes. (**A**) The A_1_R agonist CPA (10-100 nM) decreased the evoked release of ATP, whereas the A_1_R antagonist DPCPX (100 nM) was devoid of effects as such but prevented any action of CPA. (**B**) The blockade of A_2A_R in the presence of SCH58261 (50 nM) amplified the inhibitory effect of CPA and revealed a facilitatory effect of DPCPX. Data are mean SEM of n=4 different mice. *p<0.05 one-way Student’s t test *vs*. 0%; #p<0.05 two-tailed Student’s t test *vs*. absence of DPCPX; $p<0.05 *vs*. absence of SCH58261.

As shown in Fig. 2A, the selective A_1_R agonist CPA decreased the evoked release of ATP in a concentration-dependent manner, with inhibitions of 8.5±2.0% at 10 nM (n=4), 17.8±3.6% at 30 nM (n=4) and 26.1±4.4% at 100 nM (n=4). In the presence of the selective A_1_R antagonist DPCPX (100 nM), CPA (30 nM) was devoid of effects on the evoked release of ATP (p=0.077; n=4), confirming the involvement of A_1_R in the inhibitory effect of CPA on ATP release (Fig. 2A). Notably, DPCPX (100 nM) did not significantly modify the evoked release of ATP (p=0.622; n=4), indicating that endogenous adenosine does not tonically activate A_1_R to inhibit ATP release (Fig. 2A). Neither CPA (10-100 nM) nor DPCPX (100 nM) modified the basal outflow of ATP in the absence of K^+^-induced depolarization (data not shown).

To test if A_2A_R controlled A_1_R-mediated inhibition of ATP release, we next tested the ability of A_1_R to modulate ATP release upon blockade of A_2A_R. In the presence of 50 nM SCH58261, CPA at lower concentrations triggered a more robust inhibition of the evoked release of ATP, which was 20.1±2.2% at 10 nM (n=4; p=0.008 *vs*. the effect of 10 nM CPA in the absence of SCH58261), 31.3±2.3% at 30 nM (n=4; p=0.020 *vs*. the effect of 30 nM CPA in the absence of SCH58261) and 36.4±7.7% at 100 nM (n=4; p=0.267 *vs*. the effect of 100 nM CPA in the absence of SCH58261) (Fig. 2B). This more robust effect of CPA in the presence of SCH58261 only involved A_1_R activation since CPA (30 nM) was devoid of effects in the presence of 100 nM DPCPX (p=0.622; n=4) (Fig. 2B). Importantly, in the presence of SCH58261, DPCPX (100 nM) increased the evoked release of ATP by 17.7±2.7% (n=4), indicating that A_2A_R are dampening the ability of A_1_R to inhibit ATP release from hippocampal synaptosomes (Fig. 2B).

## Discussion

The present study shows that the release of ATP from nerve terminals is controlled in a dual and opposite manner by adenosine inhibitory A_1_ receptors (A_1_R) and facilitatory A_2A_ receptors (A_2A_R). The release of ATP from nerve terminals was mainly controlled by A_2A_R, which activation caused a robust increase of ATP with an efficacy far superior to that controlling other classical neurotransmitters such as glutamate [19, 20, 35], GABA [33, 36] or acetylcholine [21, 27, 37, 38]. Moreover, A_2A_R blockade revealed a tonic activation of A_2A_R bolstering the release of ATP, which was not observed when studying the evoked release of classical neurotransmitter [38–40]. In contrast, whereas A_1_R activation triggers a robust inhibition of the release of classical neurotransmitters such as glutamate [19, 20, 41] and acetylcholine [28, 39, 42, 43], A_1_R agonists caused a comparatively lower inhibition of the evoked release of ATP. Furthermore, whereas there is a constant A_1_R tonic inhibition by endogenous extracellular adenosine of the evoked release of glutamate [15, 30, 44] or acetylcholine [28, 39, 42, 43], the A_1_R antagonist DPCPX was devoid of effects on the evoked release of ATP from hippocampal nerve terminals, in contrast to the reported A_1_R-mediated inhibition of ATP release from superior cervical ganglion [45] or in cultures enriched in cholinergic amacrine-like neurons [46]. This suggests a different relative organization of A_1_R and A_2A_R to control the presynaptic release of ATP and of classical neurotransmitters in different neuronal circuits (c.f. [15, 17, 47]).

A striking particularity of the modulation by adenosine of the evoked release of ATP from hippocampal nerve terminals is the control of A_1_R-mediated inhibition by A_2A_R. In fact, we observed that the blockade of A_2A_R augmented the ability of A_1_R to inhibit ATP release, which indicates that A_2A_R curtails A_1_R function. The mechanism underlying this ability of A_2A_R to control A_1_R function may either involve the eventual release of an intermediate soluble messenger or a direct interaction between A_2A_R-A_1_R heteromers [35]. Indeed, previous neurochemical studies showed that A_2A_R and A_1_R are located in the same individual hippocampal nerve terminal [48] and that A_2A_R activation decreases A_1_R binding in hippocampal synaptosomes [22, 23]. This translates into an ability of A_2A_R to shut down inhibitory A_1_R to allow the implementation of synaptic plasticity, which would otherwise be impeded by the over-activation of A_1_R upon increased extracellular purine release at higher frequencies of nerve stimulation [4, 25]. This ability of A_2A_R to control A_1_R is further illustrated by the dependency of A_2A_R-mediated facilitation of glutamatergic transmission on an on-going A_1_R-mediated inhibition, as observed in the hippocampus [19] or in the visual [49] or neocortex [50]. Importantly, although we now observed that A_2A_R curtailed A_1_R function, the ability of A_2A_R to enhance synaptic ATP release is not dependent on A_1_R since A_2A_R enhanced ATP release and A_1_R blockade was devoid of effects. Altogether, these findings indicate that the adenosine modulation of the evoked release of ATP from hippocampal nerve terminals seems to be different from the control of the evoked release of classical neurotransmitters such as glutamate or acetylcholine: thus, the release of ATP from hippocampal nerve terminals is predominantly controlled by A_2A_R rather than A_1_R, as also previously reported for the control of neuronal ATP release in the retina [51] and ATP currents in the habenula [52].

The presently reported different modulation by adenosine of the presynaptic release of ATP and of classical neurotransmitters joins previous observations of a different calcium channel dependence [46, 53], different requirements of intensity/frequency of stimulation [4, 25] and a temporal and pharmacological dissociation of the release of ATP from the release of classical neurotransmitters in different preparations [5, 54–59]. Given the observed calcium sensitivity of the presynaptic release of ATP and previous reports that this presynaptic ATP release is vesicular in nature [2, 5, 31, 32], it remains to be determined if the presynaptic release of ATP and of classical neurotransmitter occurs from different nerve terminals (see [41]) or from different vesicles within the same nerve terminal (see [5]), as hinted by the peculiar distribution of vesicular nucleoside transporters in different synaptic vesicles [6]. Furthermore, it cannot be excluded that part of the presynaptic release of ATP might be non-vesicular, given that there are several proposed mechanisms for ATP release in different preparations [60–62]. Clearly, the mechanism of ATP release from nerve terminals remains to be adequately characterized to better understand the mechanistic basis of the observed different modulation by adenosine of the release of ATP and of classical neurotransmitters.

The presently observed ability of A_2A_R to bolster ATP release and the conclusion that the activation of A_2A_R depends on CD73-mediated ATP-derived adenosine indicates the existence of a putative feedback facilitatory loop in synapses linking ATP release/CD73 activity/A_2A_R activation. This neuronal ATP release/CD73/A_2A_R activation loop is qualitatively similar to that present in astrocytes [26] but has a different physiological meaning. In fact, the astrocytic ATP release/CD73/A_2A_R activation loop is expected to sustain a paracrine ATPergic activation of the astrocytic network (reviewed in [63]) in parallel with an adenosinergic inhibition (A_1_R-mediated) of synaptic transmission [64, 65], which contributes to implement a process of heterosynaptic depression (see [66, 67]). In contrast, the neuronal ATP release/CD73/A_2A_R activation loop is proposed to be an autocrine adenosinergic potentiation (A_2A_R-mediated) of glutamate release restricted to the ‘activated’ synapse responsible for the A_2A_R-mediated control of synaptic plasticity [13–17, 24, 68], which is selectively dependent on CD73-mediated formation of ATP-derived extracellular adenosine [11, 12, 14, 16, 32]. These different conclusions should not be viewed as antagonic but rather complementary, contributing to the implementation of salience of information encoding [69] by bolstering the activity of an ‘activated’ synapse and simultaneously decreasing the activity of surrounding synapses. This illustrates the numerous intertwined roles of the purinergic modulation system in different brain compartments [1], which further stresses the need to study the spatiotemporal gradients of extracellular ATP and adenosine in relation to the different adenosine receptors to better grasp the physiopathological roles of adenosine. This complexity is further increased by the need to recognize that apart being a substrate to ecto-nucleotidases generating adenosine, extracellular ATP also exerts direct roles as a neurotransmitter and neuromodulator [70, 71] through numerous post-synaptic and presynaptic P2X and P2Y receptors [8, 72], as championed by the group of María Teresa Miras-Portugal (e.g. [73, 74]). Importantly, it should be kept in mind that this proposed feedback facilitatory loop linking A_2A_R activation and an increased ATP release to sustain A_2A_R activation was so far only documented in purified synaptosomes and it remains to be confirmed if a similar mechanism is present in more integrated brain preparations, namely in an *in vivo* situation. Furthermore, future studies should investigate the possible contribution of this proposed feedback facilitatory loop to the synaptic dysfunction characteristic of different neuropsychiatric diseases, in view of the previously reported up-regulation of synaptic A_2A_R in numerous brain diseases (e.g. [11, 12, 18, 24, 68], as well as the established role of released ATP as a danger signal in the brain [71].

## Data Availability

The data are available from the corresponding author upon reasonable request.
